# Mast cell deficiency prevents BCR::ABL1 induced splenomegaly and cytokine elevation in a CML mouse model

**DOI:** 10.1038/s41375-023-01916-x

**Published:** 2023-05-09

**Authors:** Melanie Langhammer, Julia Schöpf, Timo Jaquet, Katharina Horn, Moritz Angel, Corinna Spohr, Daniel Christen, Franziska Maria Uhl, Tiago Maié, Henrike Jacobi, Thorsten B. Feyerabend, Julia Huber, Marcus Panning, Cassian Sitaru, Ivan Costa, Robert Zeiser, Konrad Aumann, Heiko Becker, Till Braunschweig, Steffen Koschmieder, Khalid Shoumariyeh, Michael Huber, Mirle Schemionek-Reinders, Tilman Brummer, Sebastian Halbach

**Affiliations:** 1 Institute of Molecular Medicine, ZBMZ, Faculty of Medicine, University of Freiburg, Freiburg, Germany; 2 Faculty of Biology, University of Freiburg, Freiburg, Germany; 3 Department of Hematology, Oncology, Hemostaseology, and Stem Cell Transplantation, Faculty of Medicine, RWTH Aachen University, Aachen, Germany; 4 Center for Integrated Oncology Aachen Bonn Cologne Düsseldorf (CIO ABCD), Aachen, Germany; 5 Institute of Biochemistry and Molecular Immunology, RWTH Aachen University, Aachen, Germany; 6 Spemann Graduate School of Biology and Medicine, University of Freiburg, Freiburg, Germany; 7 Department of Medicine I, Medical Center, Faculty of Medicine, University of Freiburg, Freiburg, Germany; 8 Institute for Computational Genomics, University Hospital, RWTH Aachen University, Aachen, Germany; 9 Division of Cellular Immunology, German Cancer Research Center (DKFZ), Heidelberg, Germany; 10 Department of Pathology, Institute for Surgical Pathology, Medical Center, Faculty of Medicine, University of Freiburg, Freiburg, Germany; 11 Institute of Virology, Medical Center, Faculty of Medicine, University of Freiburg, Freiburg, Germany; 12 Department of Dermatology, Medical Center, Faculty of Medicine, University of Freiburg, Freiburg, Germany; 13 German Cancer Consortium (DKTK), Partner Site Freiburg and German Cancer Research Center (DKFZ), Heidelberg, Germany; 14 Department of Pathology, University Hospital, RWTH Aachen University, Aachen, Germany; 15 Comprehensive Cancer Center Freiburg (CCCF), Medical Center, Faculty of Medicine, University of Freiburg, Freiburg, Germany; 16 Center for Biological Signalling Studies BIOSS, University of Freiburg, Freiburg, Germany

**Keywords:** Chronic myeloid leukaemia, Haematopoietic cell growth factors, Cancer microenvironment, Cancer stem cells, Translational research

## Abstract

The persistence of leukemic stem cells (LSCs) represents a problem in the therapy of chronic myeloid leukemia (CML). Hence, it is of utmost importance to explore the underlying mechanisms to develop new therapeutic approaches to cure CML. Using the genetically engineered *ScltTA/TRE-BCR::ABL1* mouse model for chronic phase CML, we previously demonstrated that the loss of the docking protein GAB2 counteracts the infiltration of mast cells (MCs) in the bone marrow (BM) of BCR::ABL1 positive mice. Here, we show for the first time that BCR::ABL1 drives the cytokine independent expansion of BM derived MCs and sensitizes them for FcεRI triggered degranulation. Importantly, we demonstrate that genetic mast cell deficiency conferred by the *Cpa3*^*Cre*^ allele prevents BCR::ABL1 induced splenomegaly and impairs the production of pro-inflammatory cytokines. Furthermore, we show in CML patients that splenomegaly is associated with high BM MC counts and that upregulation of pro-inflammatory cytokines in patient serum samples correlates with tryptase levels. Finally, MC-associated transcripts were elevated in human CML BM samples. Thus, our study identifies MCs as essential contributors to disease progression and suggests considering them as an additional target in CML therapy.

**Figure:**
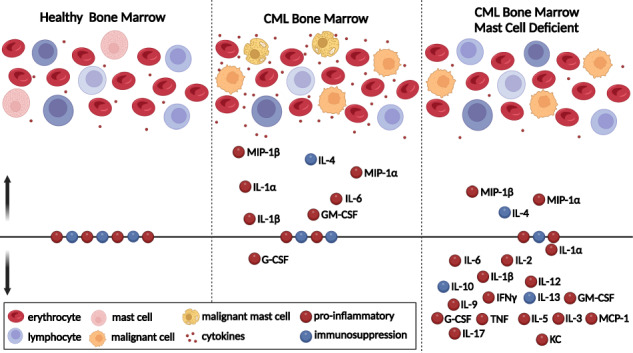
Mast cells play a key role in the pro-inflammatory tumor microenvironment of the bone marrow. Shown is a cartoon summarizing our results from the mouse model. BCR::ABL1 transformed MCs, as part of the malignant clone, are essential for the elevation of pro-inflammatory cytokines, known to be important in disease initiation and progression.

Mast cells play a key role in the pro-inflammatory tumor microenvironment of the bone marrow. Shown is a cartoon summarizing our results from the mouse model. BCR::ABL1 transformed MCs, as part of the malignant clone, are essential for the elevation of pro-inflammatory cytokines, known to be important in disease initiation and progression.

## Introduction

Chronic myeloid leukemia (CML) represents about 20% of all adult leukemia cases and is caused by a chromosomal translocation between chromosomes 9 and 22, leading to the expression of the fusion kinase BCR::ABL1 [1]. BCR::ABL1 organizes a multimeric signaling network with various components such as the docking protein GAB2 (GRB-associated-binding protein 2). GAB2 serves as an assembly platform downstream of cytokine and growth factor receptors [2]. By binding via the adaptor protein, GRB2, GAB2 amplifies the signaling into SHP2/RAS/ERK, PI3K/AKT, and STAT5 pathways, leading to survival, proliferation, and migration [2–4]. Due to its role in these oncogenic pathways, GAB2 is implicated in both solid tumors and leukemia [5]. Using GAB2 deficient mice [6], we previously showed that GAB2 serves as an important effector of the oncogenic FLT3-ITD receptor tyrosine kinase in acute myeloid leukemia (AML) [7, 8] and of BCR::ABL1 in CML [9–12]. We demonstrated that GAB2 confers resistance to clinically approved BCR::ABL1 inhibitors, including the third-generation inhibitor ponatinib [9, 12]. We also showed that GAB2 is increasingly expressed in myeloid cells from CML patients with TKI-refractory disease [12] or blast crisis [13]. Using GAB2 knock-out mice [6], we analyzed the in vivo role of GAB2 in a chronic-phase CML mouse model in which a tetracycline (tet) regulated *BCR::ABL1* transgene is expressed in hematopoietic stem cells in their native microenvironment [10, 14]. We demonstrated that GAB2 deficiency impairs disease development in a steady-state in vivo setting [10]. Surprisingly, we also detected increased numbers of mast cells (MCs) in the bone marrow (BM) and kidneys from BCR::ABL1 expressing mice. As reported previously for this mouse model [14], we observed uni- or bilateral hydronephrosis in BCR::ABL1 positive mice driven by urinary obstruction due to myeloid infiltration within the renal pelvis and ureters. Interestingly, we identified MCs as the predominant infiltrating cell type in the kidney, suggesting their contribution to hydronephrosis. Strikingly, *Gab2*^*−/−*^ mice showed neither MC infiltration in the BM or kidney nor hydronephrosis at all. This might be explained by a synergistic effect of GAB2 as a common downstream signaling effector of BCR::ABL1 and cytokine receptor signaling pathways [2]. In line with this, GAB2 has been shown to be critical for MC development and KIT signaling [15]. MCs play a role in different diseases such as allergy, as contributors to a pro-inflammatory tumor microenvironment, and as carriers of oncogenic mutations they cause mastocytosis or MC leukemia [16, 17]. Very little, however, is known about MCs in the context of CML. It was shown that MCs are increased in the BM of CML patients compared to healthy individuals and that the TKI imatinib depletes normal and neoplastic MCs in these patients [18]. However, as imatinib targets both BCR::ABL1 and KIT, it remains unclear whether BCR::ABL1 positive MCs still rely on KIT and whether the effect of this TKI reflects the inhibition of one or both targets. In addition, Askmyr et al. observed an aberrant CD25^+^ phenotype reminiscient of systemic mastocytosis in xenografts of BCR::ABL1 transduced human cord blood cells [19]. Interesting to note, basophils, which share many features and a bipotent progenitor with MCs [20], are often elevated in CML patients and used as a prognostic marker [21]. These mostly descriptive studies on MCs in BCR::ABL1 mediated transformation and our recent data from *Gab2*^−/−^ mice provided the rationale for further analysis of MCs in CML. Therefore, we aimed to analyze the role of MCs in CML in more detail. In particular, we were interested whether this MC accumulation could be driven by BCR::ABL1 itself or whether these cells reacted as bystanders to an inflammatory reaction induced by leukemic infiltrates.

Using the *ScltTA/TRE-BCR::ABL1* CML mouse model, we now show for the first time that BCR::ABL1 drives the cytokine independent expansion of BM derived mast cells (BMMCs) and sensitizes them for degranulation, IL-6 and TNF release. Importantly, by crossing in the MC-deficient *Cpa3Cre* mouse line, we discover a crucial role of MCs in CML development. We demonstrate that MC deficiency prevents BCR::ABL1 induced splenomegaly and elevation of pro-inflammatory cytokines. Furthermore, we provide supportive data from CML patients showing that splenomegaly is associated with high BM MC counts and that upregulation of pro-inflammatory cytokines in patient serum samples correlates with tryptase levels. In addition, we detected an increase of MC-associated transcripts in BM samples of CML patients.

## Methods

### Mice

*ScltTA/TRE-BCR::ABL1* mice [14] were either bred with *Gab2*^*−/−*^ mice [6] (mixed C57BL/6 x 129SV background as described previously [10]) or with *Cpa3*^*Cre/+*^ mice [22] (C57BL/6 J background). Mice were raised under specific-pathogen free conditions, with standard food and water *ad libitum*. Animal experimentation was approved by local authorities (RP Freiburg: AZ G17/69, G19/53). For genotyping primers see supplementary methods.

### Western blotting

Western Blotting was performed as described previously [23]. Antibodies are listed in supplementary methods.

### Flow cytometry

For intracellular staining, cells were fixed and permeabilized with formaldehyde and ice-cold methanol. Cell viability was assessed using 7-AAD. For immunophenotyping, BMMCs, BM and spleens cells were stained. Antibodies are listed in supplementary methods.

### β-hexosaminidase assay and ELISA

BMMCs were starved, loaded with dinitrophenylated human serum albumin (DNP-HSA)-specific IgE overnight and stimulated with DNP-HSA. Degranulation was quantified by measuring β-hexosaminidase activity and cytokine release by ELISA. Detailed procedure can be found in supplementary methods.

### Multiplex cytokine analysis

Murine BM and spleen cell lysates or serum samples from CML patients were subjected to a multiplex cytokine analysis (Bio-Plex Mouse Cytokine 23-plex or Human Cytokine 48-plex) using the Bio-Plex 200 System (Bio-Rad).

### Transcriptome analysis

Gene expression array data (Affymetrix Human Gene 1.0 ST Array) was obtained from the Gene Expression Omnibus database (accession number GSE47927) [24] for different subpopulations of cells from CML patients in chronic phase, blast crisis or from healthy individuals. Detailed procedure can be found in supplementary methods.

### Patient samples

CML patient samples from the University Hospitals Aachen and Freiburg (Supplementary Tables S2, S3) were analyzed after written informed consent according to the Declaration of Helsinki approved by the local institutional ethics committees (EK Aachen: 206/09, 391/20; EK Freiburg: 20-1253).

### Data analysis and statistics

Statistical analysis was performed using GraphPad Prism 9 and one- or two-way ANOVA and t-tests were performed as described in the figure legends. Data are presented as mean ± SEM and *p* values < 0.05 were considered statistically significant (*P < 0.05; **P < 0.01; ***P < 0.001; ****P < 0.0001).

## Results

### BCR::ABL1 drives infiltration and survival of malignant mast cells

**Fig. 1:**
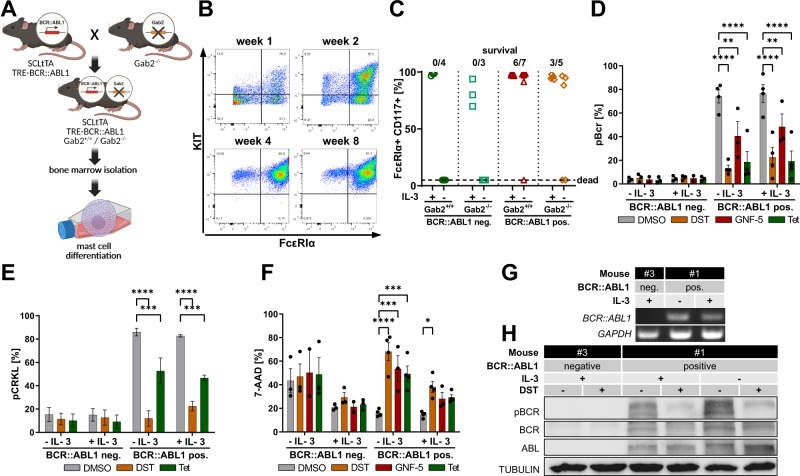
BCR::ABL1 drives differentiation and survival of mast cells. **A** Schematic overview. **B** BM was isolated and cultured in IL-3 containing media. The differentiation into MCs was monitored by flow cytometry using the MC markers FcεRIα and CD117 (KIT). Shown is one representative isolation (mouse #1, BCR::ABL1 positive). **C** BMMCs from BCR::ABL1 positive and negative mice with the indicated *Gab2* genotypes were cultured in the presence or absence of IL-3 for two weeks and monitored by flow cytometry using the MC markers FcεRIα and CD117 (KIT). **D**–**F** BCR::ABL1 negative and positive BMMCs were cultivated in standard or IL-3 containing medium and exposed to the indicated inhibitors (dasatinib=DST, 1 µM; GNF-5, 5 µM) or tetracycline (Tet, 1 µg/ml) for four days and analyzed by flow cytometry. **D** Intracellular staining for pBCR to analyze BCR::ABL1 activity. Each dot represents the BMMCs of an individual mouse and the mean of three independent performed experiments. **E** Intracellular staining for pCRKL to analyze BCR::ABL1 activity. Shown is the mean of three independent performed experiments. **F** Cell viability staining using 7-AAD. Each dot represents the BMMCs of an individual mouse and the mean of three independent performed experiments. Statistics were performed using a two way ANOVA (Fisher’s LSD test) and relevant statistically significant effects are indicated by asterisks. **G** Isolated mRNA from BMMCs (mouse #1 and #3) was reverse-transcribed into cDNA and subjected to 35 cycles of RT-PCR using BCR::ABL1 and GAPDH primers. **H** BCR::ABL1 negative and positive BMMCs were exposed to dasatinib (DST, 1 µM) for one hour and analyzed by Western Blotting using the indicated antibodies.

First, we performed BM transplantations, using BCR::ABL1 positive donor mice with different *Gab2* genotypes and myeloablative irradiated C57BL/6 N mice as recipients. (Supplementary Fig. S1A–C). Strikingly, we observed high MC counts in the BM and kidney as well as hydronephrosis in some recipients, demonstrating the cell-autonomous properties of the BCR::ABL1 positive donor cells (Supplementary Fig. S1A–C). Next, we were interested whether these cells were derived from neoplastic BCR::ABL1 transformed MC precursors or whether MCs proliferated due to secondary effects of the leukemic disease, such as enhanced growth factor expression. Therefore, we isolated BM cells from BCR::ABL1 transgenic mice and subjected them to a MC differentiation protocol by adding IL-3 to the culture medium (Fig. 1A). Differentiation over time was monitored by flow cytometry using the MC markers KIT and FcεRIα (Fig. 1B). After eight weeks in culture and onwards, over 90% of the cells stained positive for both markers. Interestingly, BMMCs from GAB2 deficient, BCR::ABL1 negative mice, displayed only a purity between 70% and 90%. Strikingly, after IL-3 deprivation only BMMCs from BCR::ABL1 positive mice survived (Fig. 1C). Furthermore, we established an intracellular flow cytometry staining of pBCR in CML cell lines (Supplementary Fig. S1D, E) and subjected this protocol to our cohort of TKI- or tet-treated BMMCs (Supplementary Fig. S1F). We observed a decrease in pBCR upon treatment with either dasatinib or the allosteric and hence highly specific BCR::ABL1 inhibitor GNF-5 [25]. This effect was mimicked by tet application, which genetically suppresses transgenic BCR::ABL1 expression (Fig. 1D; Supplementary Fig. S1G). Similar results could be obtained by using pCRKL as an alternative marker for BCR::ABL1 activity (Fig. 1E). Interestingly, the inhibitor treatments induced cell death under cytokine free conditions (Fig. 1F; Supplementary Fig. S1H). In addition, BCR::ABL1 expression was confirmed by RT-PCR and Western Blotting using anti ABL, BCR and phosphorylated BCR (pBCR) antibodies (Fig. 1G, H). Moreover, lysates from BMMCs were subjected to an array covering 110 cytokines (Supplementary Fig. S1I). Interestingly, BCR::ABL1 positive BMMCs displayed higher expression levels of most of the cytokines compared to their BCR::ABL1 negative counterparts. Only RANTES/CCL5, IL-3 and IL-23 were expressed at lower level in BCR::ABL1 positive BMMCs (Supplementary Fig. S1J).

### BCR::ABL1 enhances degranulation, cytokine release and signaling in BMMCs

**Fig. 2:**
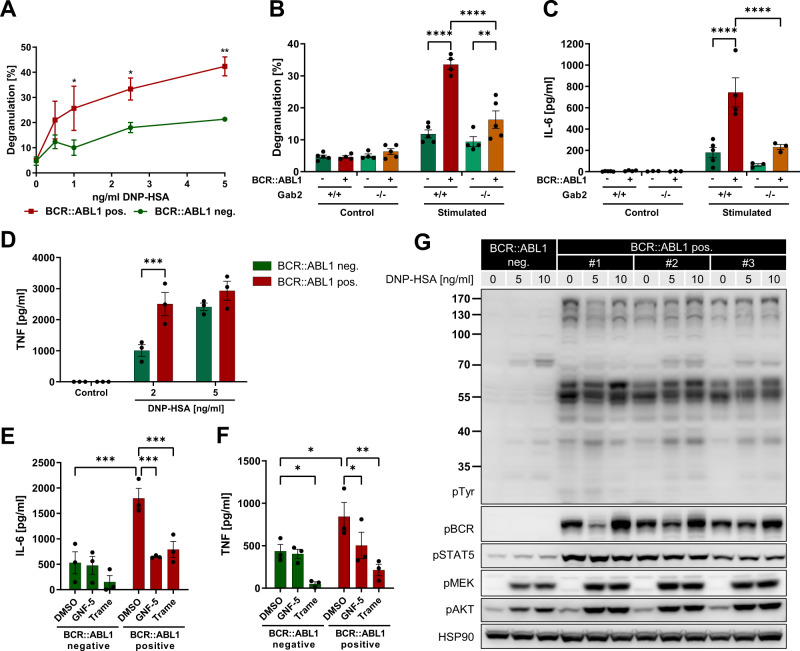
BCR::ABL1 leads to enhanced degranulation, cytokine release and signaling in BMMCs. **A** BCR::ABL1 negative (mouse #3) and positive (mouse #1) BMMCs were loaded with 50 ng/ml anti-DNP IgE overnight and stimulation with the indicated concentrations of DNP-HSA. Degranulation was assessed by β-hexosaminidase activity. **B** BCR::ABL1 negative (−) and positive (+) and *Gab2*^+/+^ or *Gab2*^−/−^ BMMCs were left untreated or loaded with 50 ng/ml anti-DNP IgE overnight and stimulated with 5 ng/ml DNP-HSA. Degranulation was assessed by β-hexosaminidase activity. **C** BCR::ABL1 negative (−) and positive (+) and *Gab2*^+/+^ or *Gab2*^−/−^ BMMCs were loaded with 150 ng/ml anti-DNP IgE overnight and either left unstimulated (Control) or stimulated for 3.5 h with 5 ng/ml DNP-HSA. The amount of secreted IL-6 was measured using an ELISA. **D** BCR::ABL1 negative (neg.) and positive (pos.) BMMCs were loaded with 150 ng/ml DNP-HSA-specific IgE overnight and either left unstimulated (Control) or stimulated for 3 h with 2 and 5 ng/ml DNP-HSA. The amount of secreted TNF was measured using an ELISA. **E**, **F** BCR::ABL1 negative and positive BMMCs were loaded with 150 ng/ml DNP-HSA-specific IgE overnight, treated with inhibitors (GNF-5, 5 µM, 60 min; trametinib=Trame, 50 nM, 30 min) and stimulated for 2 h with 2 ng/ml DNP-HSA. The amount of secreted IL-6 (**E**) and TNF (**F**) was measured using an ELISA. **A**–**F** Statistics were performed using a two-way ANOVA (Fisher’s LSD) and relevant statistically significant effects are indicated by asterisks. **B**–**F** Each dot represents the BMMCs of an individual mouse and the mean of three independent performed experiments conducted in triplicates. **G** BCR::ABL1 negative and positive BMMCs were loaded with 150 ng/ml DNP-HSA-specific IgE overnight, stimulated with the indicated concentrations of DNP-HSA and analyzed by Western Blotting using the indicated antibodies.

Next, we assessed MC functionality and signaling by degranulation assays and cytokine release. To this end, we loaded BMMCs with DNP-HSA-specific IgE, stimulated them with antigen (DNP-HSA) and measured β-hexosaminidase activity to quantify degranulation. First, we titrated the amount of IgE and DNP-HSA (Fig. 2A; Supplementary Fig. S2A), followed by an analysis of more samples including BMMCs from GAB2 deficient mice (Fig. 2B; Supplementary Fig. S2B). Interestingly, BCR::ABL1 positive BMMCs were more sensitive towards antigen stimulation and displayed a stronger degree of degranulation. Strikingly, GAB2 deficient and BCR::ABL1 positive BMMCs showed only a marginal elevation in their degranulation levels compared to their BCR::ABL1 negative counterparts. In line with these results, we observed higher levels of secreted IL-6 and TNF in BCR::ABL1 positive BMMCs after DNP-HSA stimulation (Fig. 2C, D; Supplementary Fig. S2C, D). Again, GAB2 deficiency reduced the elevation of IL-6 secretion in BCR::ABL1 positive BMMCs. In addition, treatment of BMMCs with GNF-5 or the MEK inhibitor trametinib counteracted the BCR::ABL1 induced up-regulation of IL-6 and TNF (Fig. 2E, F). As GAB2 deficiency prevented BCR::ABL1 positive BMMCs from secreting elevated IL-6 levels, we were interested whether GAB2 promotes IL-6 secretion also in a human CML model. To this end, we analyzed IL-6 secretion in the cell line K562, in which GAB2 expression was suppressed by inducible shRNAs (Supplementary Fig. S2E, F). Strikingly, GAB2 depletion also reduced IL-6 secretion in this model. As GAB2 amplifies the signaling from BCR::ABL1 to PI3K and via SHP2 to the ERK pathway [2], we performed inhibitor experiments targeting BCR::ABL1, the PI3K and the ERK pathway (Supplementary Fig. S2G, H). As expected, the inhibition of BCR::ABL1 by imatinib or dasatinib suppressed IL-6 secretion. In line with our data from BMMCs, the inhibition of the ERK pathway by targeting SHP2 with SHP099 or MEK with trametinib also reduced IL-6 secretion, whereas the treatment with the dual PI3K/mTOR inhibitor BEZ-235 strongly increased the secretion of IL-6. Next, we analyzed FcεRI signaling of BCR::ABL1 positive BMMCs after stimulation with DNP-HSA by Western Blotting (Fig. 2G). Independent of DNP stimulation, we already observed an increase in BCR, STAT5 and overall tyrosine phosphorylation in BCR::ABL1 positive compared to BCR::ABL1 negative BMMCs, while upregulation of pMEK and pAKT levels still required FcεRI activation.

### Mast cell deficiency impairs CML development in *ScltTA/TRE-BCR::ABL1* mice

**Fig. 3:**
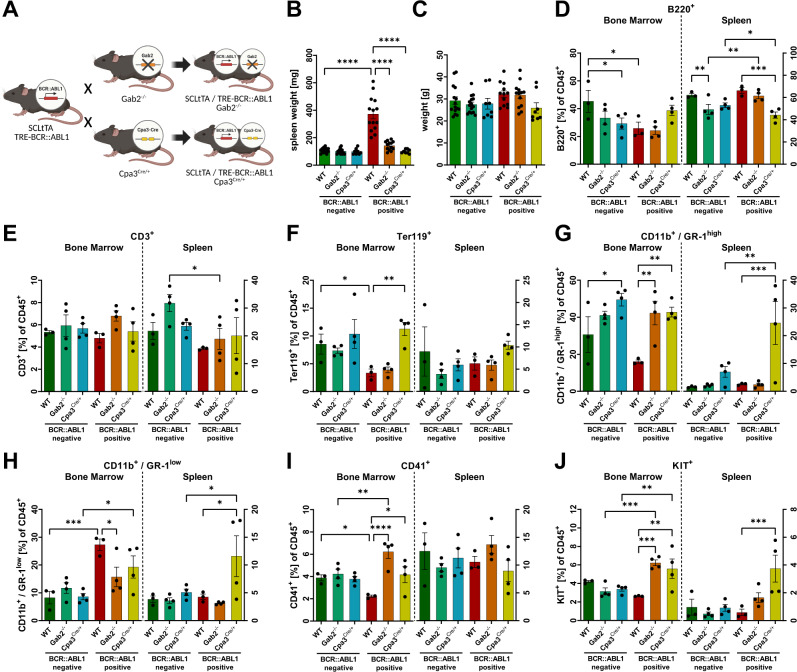
Mast cell deficiency protects against the development of leukemia symptoms in *ScltTA/TRE-BCR::ABL1* transgenic mice. **A** *ScltTA/TRE-BCR::ABL1* double transgenic mice were either crossed with *Gab2*^*−/−*^ or *Cpa3*^*Cre/+*^ mice. **B** Spleen weight of mice 60 days after tetracycline withdrawal. **C** Weight of mice 60 days after tetracycline withdrawal. **D**–**J** Composition of BM and spleen cells of mice 60 days after tetracycline withdrawal assessed by flow cytometry for the indicated markers. Each dot represents the biopsy of one individual mouse. All statistics were performed using a one-way ANOVA (Fisher’s LSD test) and relevant statistically significant effects are indicated by asterisks.

To further investigate the role of BCR::ABL1 in MCs in CML pathogenesis, we applied the MC-deficient *Cpa3*^*Cre/+*^ mouse line [22] in two genetic approaches. First, we retrovirally transduced BM from these mice using vectors expressing GFP, either singly or in combination with BCR::ABL1. The BM was then transplanted into C57BL/6 J recipients that were analyzed 25 days later (Supplementary Fig. S3A). We observed expansion of neutrophilic cells as shown by an increase of immature, CD11b^+^ / GR-1^+^ cells in BM and spleen from mice that received BCR::ABL1 positive BM compared to BCR::ABL1 negative controls. This was accompanied by a decrease of B220^+^ cells in the CML mice (Supplementary Fig. S3C, D). Interestingly, the increase of immature neutrophilic cells (CD11b^+^ / GR-1^low^) was significantly lower in BM from mice transplanted with BCR::ABL1 positive *Cpa3*^*Cre/+*^ cells. In the spleen, an unsignificant trend was pointing in the same direction. Spleen weight was elevated in the BCR::ABL1 positive groups, but we did not observe a difference between the WT and *Cpa3*^*Cre/+*^ group (Supplementary Fig. S3E). The percentage of LSK cells was not altered between the groups (Supplementary Fig. S3F, G). Next, we analyzed the mRNA expression of IL-1β and TNF by qRT-PCR in the BM of these mice (Supplementary Fig. S3H, I). Interestingly, the expression of both cytokines was significantly lower for the BCR::ABL1 positive *Cpa3*^*Cre/+*^ condition compared to the WT control. As we have used WT recipients here, we cannot exclude that residual MCs from the recipients were able to re-expand in this model. Therefore, we next implemented a transgenic approach to completely abolish MC development. In this approach, we crossed *Cpa3*^*Cre/+*^ mice with *ScltTA/TRE-BCR::ABL1* mice. In addition, we included mice lacking GAB2 in our analysis (Fig. 3A). Mice were sacrificed 60 days after BCR::ABL1 induction by tet withdrawal, and BM and spleen were analyzed. BCR::ABL1 positive mice displayed enlarged spleens with up to 5-fold increase in spleen weight compared to WT mice in keeping with a CML phenotype. Strikingly, GAB2 or MC deficient animals within the BCR::ABL1 positive group showed no signs of splenomegaly (Fig. 3B), which suggested that the CML phenotype required the presence of MCs. Body weight was not altered between the groups (Fig. 3C). Next, we assessed the compositions of the cells by surface markers. We observed a significant decrease in B220+, Ter119+ and CD41+ cells and an expansion of immature, CD11b^+^ / GR-1^low^ cells in BM from BCR::ABL1 positive mice compared to WT mice (Fig. 3D–I). The cellularity from the spleen of these mice was not altered significantly. Interestingly, there was no decrease in B220+, Ter119+ and CD41+ cells in the BM of BCR::ABL1 positive *Cpa3*^*Cre/+*^ mice and immature cells showed only a mild expansion. The latter was also observed for the BM of BCR::ABL1 positive *Gab2*^*−/−*^ mice. In addition, we observed an expansion of KIT positive cells in BM and spleen cells from BCR::ABL1 positive mice, but only in those which were deficient for MCs or GAB2 (Fig. 3J).

### Mast cell deficiency prevents BCR::ABL1 induced cytokine elevation in BM and spleen from *ScltTA/TRE-BCR::ABL1* mice

**Fig. 4:**
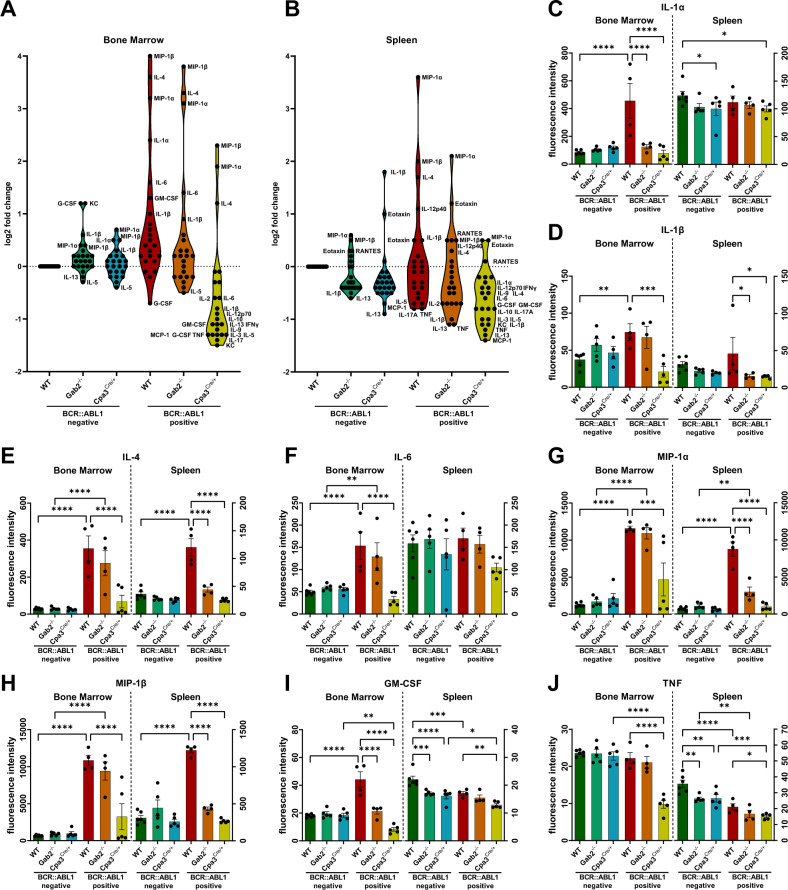
Mast cell deficiency blocks BCR::ABL1 induced cytokine elevation in bone marrow and spleen from *ScltTA/TRE-BCR::ABL1* transgenic mice. **A**–**J** Total cell lysates from BM and spleen of mice 60 days after tetracycline withdrawal were subjected to a multiplex cytokine analysis. **A**, **B** Shown is a violin blot, data is normalized to WT control and log2 transformed. **C**–**J** Shown is the flouresecense intensity of individual cytokines. Each dot represents the biopsy of one individual mouse. All statistics were performed using a one-way ANOVA (Fisher’s LSD test) and relevant statistically significant effects are indicated by asterisks.

As cytokines play a key role in the pathogenesis of CML [24], we next assessed their expression levels in BM and spleen from *ScltTA/TRE-BCR::ABL1* mice either crossed with *Cpa3*^*Cre/+*^ or *Gab2*^−/−^ mice (Fig. 4; Supplementary Fig. S4). Mice were sacrificed 60 days after induction of BCR::ABL1 and BM and spleen were isolated. We observed a significant elevation of IL-1α, IL-1β, IL-4, IL-6, MIP1-α, MIP1-β and GM-CSF in the BM of BCR::ABL1 positive mice compared to WT control (Fig. 4; Supplementary Fig. S4). Interestingly, there was no BCR::ABL1 induced upregulation of IL-1α and GM-CSF visible in GAB2 deficient BM (Fig. 4A, C, I). Even more remarkable was the comparison with BCR::ABL1 positive *Cpa3*^*Cre/+*^ mice. IL-4, MIP-1α, and MIP-1β were only increased in some samples and to a lesser extent compared to BCR::ABL1 positive WT mice (Fig. 4A, E, G, H). Furthermore, IL-1α, IL-1β, IL-6, and GM-CSF were even reduced compared to WT (Fig. 4A, D, F, I). In addition, we detected a range of cytokines, which were not or only slightly altered by BCR::ABL1, but again downregulated in BM from BCR::ABL1 positive *Cpa3*^*Cre/+*^ mice. Among these cytokines were TNF, IFNγ, IL-2, IL-3, IL-5, IL-9, IL-10, IL-12, IL-13, IL-17, KC, and MCP-1 (Fig. 4A, J; Supplementary Fig. S4). Next, we analyzed the spleens of these mice and observed similarities but also differences compared to the BM. MIP1α, MIP1β, IL-1β and IL-4 were again upregulated in BCR::ABL1 positive cells compared to WT. These cytokines were significantly lower or not increased in samples from *Cpa3*^*Cre/+*^ mice and, in contrast to BM, also from *Gab2*^*−/−*^ mice (Fig. 4B, D, E, G, H). In line with our data from BM, we detected a strong downregulation of cytokines in BCR::ABL1 positive *Cpa3*^*Cre/+*^ samples compared to WT control (Fig. 4B; Supplementary Fig. S4).

### MC-associated transcripts, tryptase and pro-inflammatory cytokines were elevated in CML patient samples

**Fig. 5:**
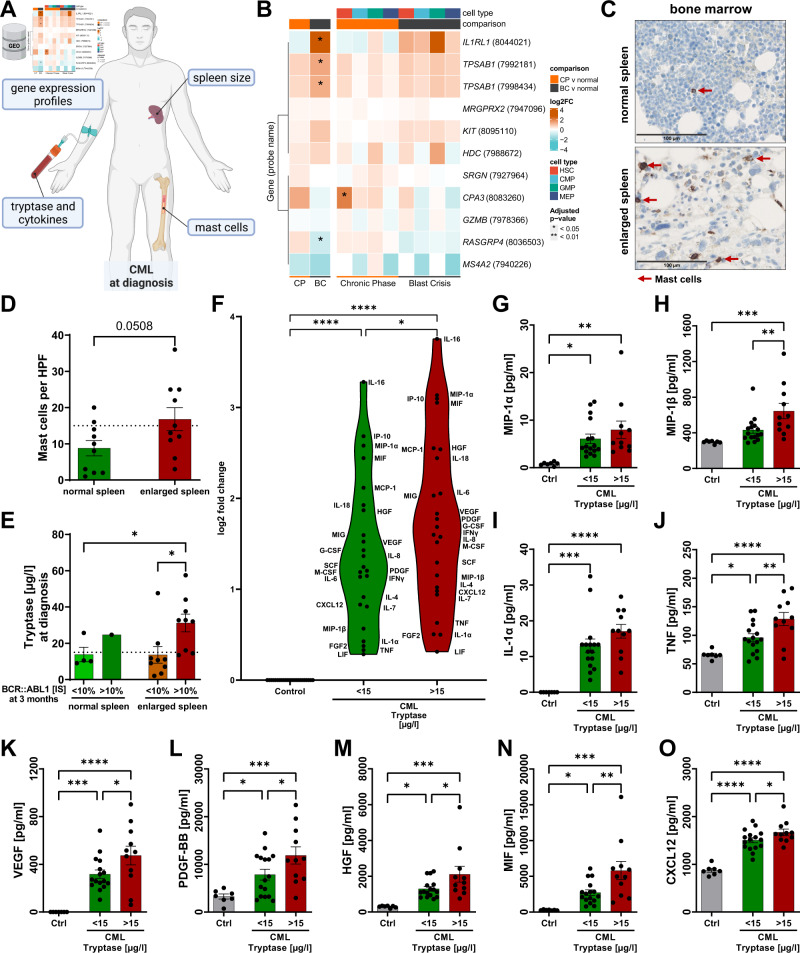
MC-associated transcripts, tryptase and pro-inflammatory cytokines were elevated in CML patient samples. **A** Scheme summarizing the patient data from CML patients. **B** Transcriptome analysis of MC associated genes (GEO accession GSE47927). Note: For TPSAB1, two distinct probe IDs were available. *P*-values were corrected for multiple testing with the Benjamini-Hochberg procedure. **C** Shown is an exemplary MCT staining in the BM of a CML patient with and without an enlarged spleen. MCs are highlighted with red arrows. **D** Quantification of MCT-stained MCs in the BM of CML patients with and without an enlarged spleen. **E** Tryptase levels in the serum of CML patients with and without an enlarged spleen at diagnsosis. Patients are grouped according to treatment response. **F**–**O** Serum samples from CML patients at diagnosis and healthy controls were subjected to a multiplex cytokine analysis. **F** Shown is a violin blot, primary data is normalized to healthy control and log2 transformed. **G**–**O** Shown is the calcualted concentration of individual cytokines. Each dot represents the biopsy of one individual. Statistics were performed using an unpaired *t*-test (two-tailed) (**D**), a two-way ANOVA (Fisher’s LSD test) (**F**) and a one-way ANOVA (Fisher’s LSD test) (**G**–**O**) and relevant statistically significant effects are indicated by asterisks.

Finally, we analyzed gene expression profiles, spleen size, tryptase and cytokines levels from CML patients (Fig. 5A). First, we characterized the gene expression profiles of MC associated genes in different subpopulations obtained from CML patients in chronic phase and blast crisis or from healthy individuals (GEO accession number GSE47927) (Fig. 5B). Interestingly, most of these genes were upregulated in chronic phase, particularly in the HSC and GMP subpopulations. This upregulation was even more pronounced in blast crisis for *IL1RL1*, *TPSAB1*, *KIT*, and *HDC*, while *CPA3*, *RASGRP4*, and *MS4A2* were downregulated in this setting. Next, we stained MCs by MC tryptase (MCT) in BM biopsies of 20 CML patients at diagnosis (Fig. 5C; Supplementary Table S2). Interestingly, we detected a strong trend indicating that high MC counts correlate with splenomegaly (Fig. 5D). Furthermore, we determined the serum levels of tryptase and cytokines at diagnosis in an independent cohort of 27 CML patients (Fig. 5E-O; Supplementary Fig. S5 and Table S3). Strikingly, patients presenting with enlarged spleens in combination with elevated tryptase levels at diagnosis have a higher risk for an insufficient therapy response, determined by *BCR::ABL1*/ABL1 ratios (*BCR::ABL1 [IS])* after three month of TKI therapy (Fig. 5E). Moreover, we detected an upregulation of pro-inflammatory cytokines in CML samples compared to healthy controls (Fig. 5F–O; Supplementary Fig. S5). Remarkably, this upregulation was significantly more pronounced in the serum of patients with higher tryptase levels. This was especially the case for MIP1β, TNF, VEGF, PDGF, HGF, MIF and CXCL12 (Fig. 5G–O).

## Discussion

MCs play a key role in allergic responses, in the pathogenesis of immunologic disorders and are implicated in cancer due to their contribution to a pro-inflammatory tumor microenvironment [16, 17]. Here, we show for the first time that MCs play an important role in a chronic phase CML mouse model for this disease. First, we observed infiltration of MCs in the BM and kidney of mice, which were transplanted with BCR::ABL1 positive BM cells (Supplementary Fig. S1A–C). This observation was in line with our previous results from the primary setting of this mouse model [10] and demonstrates that these alterations were caused by the BCR::ABL1 positive donor cells. Furthermore, we show that GAB2 deficiency protects from the infiltration of MCs in these organs (Supplementary Fig. S1A, B). This might be explained by the fact that GAB2 signals downstream of both BCR::ABL1 and KIT, which is essential for MC development [15]. In addition, we demonstrated that BCR::ABL1 can drive the expansion of MCs under cytokine free conditions (Fig. 1C). Consequently, these BCR::ABL1 positive BMMCs were sensitive towards inhibition or genetic ablation of BCR::ABL1 (Fig. 1D–F). This is further supported by our observation that BCR::ABL1 positive BMMC displayed constitutive and enhanced STAT5 signaling (Fig. 2G), a critical driver of MC development and survival [26]. Our findings agree with earlier reports showing that BCR::ABL1 transduced hematopoietic progenitors and human CML xenografts can generate MCs [19, 27–29] or MC-related basophils as in the human CML line KU812 [30]. Consistent with this, we detected an upregulation of MC-associated genes in BM samples from CML patients, particularly in the pathologically relevant HSC and GMP subpopulations, compared to healthy individuals (Fig. 5B). With the exception of a few, such as *RASGRP4*, which was downregulated, upregulation was more pronounced in blast crisis than in chronic phase, suggesting MC expansion along with disease progression. The fact that *RASGRP4* is downregulated supports our hypothesis of the development of a malignant MC pool, as functionally inactive *RASGRP4* mutants were also found to be expressed in patients with mastocytosis and MC leukemia [31]. Next, we assessed MC functionality of the murine BMMCs by degranulation assays and cytokine release. Importantly, BCR::ABL1 positive cells were more sensitive towards antigen stimulation and displayed a stronger degranulation and higher levels of secreted IL-6 and TNF compared to BCR::ABL1 negative controls (Fig. 2A–D). This suggests a positive influence of BCR::ABL1 on the proximal FcεRI signaling cascade. The resulting elevated degranulation and release of pro-inflammatory cytokines could explain the hydronephrosis observed in our CML mouse model. Commensurate with these results, GAB2 deficient cells from BCR::ABL1 transgenic mice showed neither elevated degranulation levels nor increased IL-6 release (Fig. 2B, C), which agrees with the role of GAB2 downstream of FcεRI [32]. This is further supported by earlier studies with an independently generated *Gab2*^*−/−*^ mouse strain showing less degranulation and cytokine gene expression after antigen stimulation [32]. We confirmed the relevance of GAB2 for cytokine production in the human CML cell line K562, in which its depletion also reduces IL-6 expression (Supplementary Fig. S2G, F). As GAB2 broadens the oncogenic signals from BCR::ABL1 into the ERK and PI3K pathway [2], we performed inhibitor experiments in BMMC and K562 cells (Fig. 2E, F; Supplementary Fig. S2G, H). Interestingly, the clinically applied MEK inhibitor trametinib strongly downregulates IL-6 and TNF levels, pointing towards an implication of the ERK pathway (Fig. 2E, F). Notably, we detected a stronger increase in MEK phosphorylation in BCR::ABL1 positive compared to BCR::ABL1 negative BMMCs after DNP-HSA stimulation (Fig. 2G). In contrast, the treatment with BEZ-235 increases IL-6 expression in K562 cells (Supplementary Fig. S2G, H), suggesting an inhibitory role of the PI3K pathway, for example by reducing its negative crosstalk with the ERK pathway [33].

Encouraged by these results, we further probed the role of MCs in CML by using *Cpa3*^*Cre/+*^ mice characterized by genetically induced MC deficiency [22]. Mice from this model display a normal immune system, apart from the lack of MCs and reduced basophils. In a first attempt, we used a retroviral model in which BCR::ABL1 was introduced into BM cells from *Cpa*^*Cre/+*^ mice and then transplanted into WT recipients (Supplementary Fig. S3). Interestingly, the loss of MCs in the BM of mice transplanted with BCR::ABL1-positive *Cpa3*^*Cre/+*^ donor cells attenuates the increase in immature, CD11b^+^/GR-1^low^, cells compared to their MC-competent counterparts (Supplementary Fig. S3C). As we did not observe any impact of MC deficiency on spleen weight, which represents one of the critical prognostic markers of the Sokal score [34], we switched our approach and crossed the *Cpa*^*Cre*^ allele into the *ScltTA/TRE-BCR::ABL1* model. Remarkably, and in contrast to the retroviral model, BCR::ABL1-positive MC-deficient mice showed no signs of splenomegaly (Fig. 3B). These at first glance controversial results might be explained by the main differences between retroviral transduction/transplantation and genetically engineered mouse models. In the latter, the *BCR::ABL1* transgene is expressed in hematopoietic stem cells in their native microenvironment, allowing analysis under steady-state conditions [10, 14]. This circumvents the main disadvantages of the retroviral model, such as the variability in BCR::ABL1 overexpression and disease phenotype between recipients. Furthermore, the retroviral model shows rapid disease onset with fatal outcome shortly after transplantation and hence rather resembles an acute leukemia, while the genetic model recapitulates more the chronic phase of the disease [35]. In addition, there is also the possibility that BCR::ABL1 was expressed only in MCs of the transgenic but not of the retroviral mouse model.

Importantly and in line with the data from the genetic model, we observed a correlation of splenomegaly and high BM MC counts in our patient cohort (Fig 5D). As shown in our previous study [10], BCR::ABL1 positive GAB2 deficient mice, which were also included in this study, displayed a similar phenotype as the MC deficient mice (Fig. 3B). Interestingly, we observed only a mild expansion of immature cells in the BM of BCR::ABL1 positive MC and GAB2 deficient mice compared to their proficient counterparts (Fig. 3H). Cytokines in the BM niche are often deregulated in CML and implicated in disease progression. In particular, pro-inflammatory cytokines, such as IL-1α [36], IL-1β [37], IL-6 [38, 39], TNF [36, 38] and MIP-1β [36], are upregulated in the serum or BM of CML patients. This is supported by our own serum analysis of a small patient cohort, in which we also detected significantly higher levels of pro-inflammatory cytokines compared to healthy individuals (Fig 5F–O; Supplementary Fig. S5 and Table S3). Therefore, we analyzed the cytokine profile of our mouse cohort. In line with a previous study by Zhang et al. [36], we now show elevated levels of IL-1α, IL-1β, IL-4, IL-6, TNF, GM-CSF, MIP-1α and MIP-1β in the BM of *ScltTA/TRE-BCR::ABL1* mice (Fig. 4A, C–J). Strikingly, we were not only able to confirm the results, but also show that the loss of MCs counteracts the BCR::ABL1-induced increase of these cytokines or even leads to a downregulation compared to WT. In addition, we presented similar data for the spleen, as here IL-1β, IL-4, MIP-1α and MIP-1β were upregulated by BCR::ABL1 and again not altered or downregulated with the loss of MCs (Fig. 4B, D, G, H). Moreover, we demonstrated in our patient cohort that higher serum levels of tryptase correlate with significantly higher levels of MIP1β, TNF, VEGF, PDGF, HGF, MIF and CXCL12 (Fig. 5F–O). Taken together, this suggests that BCR::ABL1 positive MCs either express these pro-inflammatory cytokines themselves or at least stimulate other cells to do so. This is further supported by our observation that these cytokines are also upregulated in BCR::ABL1 driven BMMC (Supplementary Fig. S1J). The upregulation of these cytokines is of particular interest as a pro-inflammatory environment provides a selective advantage for leukemic stem cells (LSCs) [40]. Several studies demonstrated that IL-1α/β [41–43], IL-6 [44, 45], GM-CSF [46] and MIP-1α [47, 48] exert positive regulatory effects to expand primitive CML cells. Furthermore, IL-4 has been shown to maintain survival of CML cells on TKI inhibition [49] and is known to antagonize MHC-II and CIITA expression [50, 51], which promotes immune evasion [52]. By contrast, chronic exposure to MIP-1α and IL-1α/β exhausts normal HSC [53–55]. Furthermore, we observed that a range of cytokines such as TNF, IFNγ, IL-2, IL-3, IL-5, IL-9, IL-10, IL-12, IL-13, IL-17, KC and MCP-1 were downregulated in BM from BCR::ABL1 positive *Cpa3*^*Cre/+*^ mice, suggesting that BCR::ABL1 positive MCs are also involved in the regulation of these cytokines (Fig. 4A; Supplementary Fig. S4). This is of particular interest as some of these cytokines are described to support CML progression and therapy resistance. For example, TNF supports the survival of CML stem and progenitor cells [56] and IFNγ reduces the sensitivity towards TKIs [57]. We were also able to show, that the loss of GAB2, as an important signaling amplifier in MCs, counteracts the BCR::ABL1 induced elevation of some of these cytokines such as IL-1α in the BM and MIP-1α, MIP-1β and IL-4 in the spleen (Fig. 4A–C, E, G, H). Finally, we demonstrated in our patient cohort that enlarged spleens in combination with elevated serum tryptase levels correlate with a diminished response to therapy (Fig. 5E). This supports the concept of the modified EUTOS score, in which basophils are replaced with serum tryptase. This EUTOS-T score was evaluated in a large patient cohort and shows a more accurate prediction of treatment response [58].

In summary, we demonstrate that BCR::ABL1 can drive the expansion of murine MCs and that these BCR::ABL1 transformed MCs, as part of the malignant clone, are essential for the disease associated development of splenomegaly and for the elevation of pro-inflammatory cytokines, known to be important in disease initiation and progression. These data are supported by our CML patient analyzes in which we show that splenomegaly is associated with high BM MC counts and that upregulation of pro-inflammatory cytokines in patient serum samples correlates with tryptase levels. Thus, our study suggests that MCs play an essential role in CML and might serve as an additional target in the clinic. This is of particular relevance, as BCR::ABL1 positive MCs might be resistant against TKIs in the cytokine rich BM niche. This is supported by our observation that IL-3 protects BCR::ABL1 positive BMMCs from TKI induced cell death.

As pro-inflammatory cytokines are known to be important for many other cancer entities, our data also invites for the evaluation on the role of MCs beyond CML. In addition, this data and our previous work on GAB2 also highlights the possibility that GAB2, as a common player in BCR::ABL1 and MC signaling could serve as an additional target in the treatment of CML.

## Supplementary information

Supplemental Information

## Data Availability

All data supporting the findings of this study are available within the article and its supplementary information and from the corresponding author upon reasonable request.
