# Supplementary material for: Mast cell deficiency prevents BCR::ABL1 induced splenomegaly and cytokine elevation in a CML mouse model
Source: Leukemia. 2023 May 9;37(7):1474–84. doi: 10.1038/s41375-023-01916-x (PMC10317838; doi:10.1038/s41375-023-01916-x)
Supplement: Supplementary file 1 — Supplemental Information [file 41375_2023_1916_MOESM1_ESM.pdf]

Supplemental Information for

Mast cell deficiency prevents BCR::ABL1 induced splenomegaly and cytokine elevation in a CML mouse model

Melanie Langhammer, Julia Schöpf, Timo Jaquet, Katharina Horn, Moritz Angel, Corinna Spohr, Daniel Christen, Franziska Maria Uhl, Tiago Maié, Henrike Jacobi, Thorsten B. Feyerabend, Julia Huber, Marcus Panning, Cassian Sitaru, Ivan Costa, Robert Zeiser, Konrad Aumann, Heiko Becker, Till Braunschweig, Steffen Koschmieder, Khalid Shoumariyeh, Michael Huber, Mirle Schemionek-Reinders, Tilman Brummer and Sebastian Halbach

Supplemental Material and Methods

Mouse genotyping

Mice were genotyped by PCR using the following primer pairs:

Allele	Primer	Sequence
TetO-BCR-ABL	BCR-ABL sense	GAGCGTGCAGAGTGGAGGGAGAACA
	BCR-ABL antisense	GGTACCAGGAGTGTTTCTCCAGACTG
ScltTA	SCL	AGAACAGAATTCAGGGTCTTCCTT
	oIMR 5987 Tet	TTTCGATCTGGACATGTTGG
GAB2	GAB2 WT 1	AACTACGTGCCCATGAACCCAGGTTC
	GAB2 WT 2	AGATGGGGCAAAAAGTTGGCTTACC
	GAB2 MUT 1	CGCCTACCGGTGGATGTGGAATGTG
	GAB2 MUT 2	CCCTGGGTTATCATTCTCCGCTGGG
CPA3	CPA3 Common	GGACTGTTCATCCCCAGGAACC
	CPA 3 wt	CTGGCGTGCTTTTCATTCTGG
	CPA 3 mut	GTCCGGACACGCTGAACTTG

Antibodies

**Antigen**	**Application**	**Fluorophore**	**Order number/ Clone**	**Company**
B220	Flow Cytometry	PE Cy5	103222 / RA3-6B2	BioLegend
CD117 (KIT)	Flow Cytometry	PB	105820 / 2B8	BioLegend
CD11b	Flow Cytometry	PE Cy7	101226 / M1/70	BioLegend
CD3	Flow Cytometry	PB	100214 / 17A2	BioLegend
CD41	Flow Cytometry	APC Cy7	133914 / MWReg30	BioLegend
CD45	Flow Cytometry	Alexa Fluor 700	103128 / 30-F11	BioLegend
FcεRIa	Flow Cytometry	PE	134308 / MAR-1	BioLegend
GR1	Flow Cytometry	PE Cy5	108410 / RB6-8C5	BioLegend
pCRKL Y207	Flow Cytometry	-	3181	Cell Signaling
Rabbit	Flow Cytometry	Alexa Fluor 488	406416	BioLegend
Ter119	Flow Cytometry	APC	116212 / TER-119	BioLegend
ABL	Western Blotting	-	2862	Cell Signaling
pAKT S473	Western Blotting	-	4060	Cell Signaling
AKT	Western Blotting	-	9272	Cell Signaling
pBCR Y177	Western Blotting<br>Flow Cytometry	-	3901	Cell Signaling
BCR	Western Blotting	-	3902	Cell Signaling
pERK	Western Blotting	-	4376	Cell Signaling
ERK	Western Blotting	-	9102	Cell Signaling
GAB2	Western Blotting	-	3239	Cell Signaling
pMEK S221	Western Blotting	-	2338	Cell Signaling
pSTAT5 Y694	Western Blotting	-	9351	Cell Signaling
pTyr 4G10	Western Blotting	-	96215	Cell Signaling
GAPDH	Western Blotting	-	ab9489	Abcam
HSP90	Western Blotting	-	4877	Cell Signaling
TUBULIN	Western Blotting	-	SC-23948	Santa Cruz

Transplantation of bone marrow from *ScltTA/TRE-BCR::ABL1* mice

Isolated bone marrow (BM) from *SclTA/TRE-BCR::ABL1* mice was transplanted into 8-week old C57BL/6N mice (Janvier Labs). Recipients underwent myeloablative body irradiation (2 × 5 Gy, 4h apart) followed by intravenous injection of 5×10^6 BM cells. The donors received tetracycline hydrochloride until BM isolation leading to BCR::ABL1 expression only in the newly developing hematopoietic system. Only sex-matched donor-recipient pairs were used.

Hematological analysis of murine samples

Formalin fixed and paraffin embedded tissue sections from murine kidney and de-calcified BM were stained with Giemsa according to standard protocols, as described elsewhere.[1]

Cells

KBM5 and KBM5-STI cells were kindly provided by Michael Andreeff and Miloslav Beran, Houston, USA. K562 cells were a kind gift from Mark Guthridge, Melbourne, Australia. All cells were cultivated under water vapor saturated atmosphere at 37°C and 5% CO_2. K562 cells were maintained in RPMI-based medium. KBM-5 cells were maintained in IMDM-based medium (PAN-Biotech) supplemented with 10% FBS. BMMCs were cultured in IMDM-based (PAN-Biotech) growth medium supplemented with 20% FBS (Biochrom) and IL-3 (supernatant from X63-IL-3 cells).

Generation of bone marrow-derived mast cells (BMMCs)

BM cells were isolated by flushing femora and tibiae with medium containing IL-3. The cells were centrifuged (5 min at 2000g) and medium was changed every other day for four weeks. Cells were analyzed every week by flow cytometry using antibodies against KIT and FcεRIa.

Reverse transcription of mRNA and PCR

RNA was isolated using the RNA Mini Kit (Analytik Jena AG). Reverse Transcription and RT PCR was performed using the RevertAid First Strand cDNA Synthesis Kit (Thermo Fisher Scientific).

Cytokine array

BMMC were lysed and subjected to the Proteome Profiler Mouse XL Cytokine Array (R&D Systems) according to the manufacturers protocol.

Transcriptome analysis

Gene expression array data (Affymetrix Human Gene 1.0 ST Array) was obtained from the Gene Expression Omnibus database (accession number GSE47927) for different subpopulations of cells from patients with CML in chronic phase, blast crisis or from healthy individuals. MPP subpopulations were excluded from this analysis. Samples were preprocessed and analysed with R (version 4.2.1) and R Bioconductor packages GEOquery, Biobase, data.table and limma as well as in-house scripts. Data was normalized with the limma function "normalizeBetweenArrays" using method "cyclicloess" and otherwise default settings. Differential expression analysis was done with limma by fitting a linear model for each comparison and estimating the statistics with empirical Bayes moderation. P-values were corrected for multiple testing with the Benjamini-Hochberg procedure. Analysis was performed on the entire dataset but only MC associated genes were kept for visualization.

Inhibitor and tetracycline treatment

Imatinib, Dasatinib, GNF-5, BEZ-235, SHP099 and Trametinib were purchased from Selleckchem and Tetracycline from Sigma.

β-hexosaminidase assay

BMMCs were starved, loaded with α-DNP IgE SPE-7 (Sigma-Aldrich) over night and stimulated with DNP-HSA (Sigma-Aldrich) for 30 minutes in Tyrode's Buffer (10 mM HEPES, 130 mM NaCl, 5mM KCL, 1.4 mM $CaCl_2$, 1 mM $MgCl_2$, 5.6 mM Glucose, 0.1% BSA in dH_20) at 37°C. The reaction was stopped on ice, the supernatant collected and the pellet lysed with 0.5% NP-40. Lysate and supernatant were plated on a 96-well-plate in duplicates and mixed with Substrate Solution (1.3 mg/ml p-nitrophenyl-N-acetyl-β-D-glucosaminide in 0.1 M Na-citrate pH 4.5). After 90 minutes of incubation at 37°C, 150 µl Glycine Solution (0.2 M glycine in dH_2O pH 10.7) was added to stop the reaction. The absorbance at 405 nm was measured and the percentage of degranulation calculated: % Degranulation = OD405 supernatant / (OD405 supernatant + OD405 lysate).

ELISA

BMMCs were loaded with α-DNP IgE SPE-7 (Sigma-Aldrich) and starved overnight. The next day the cells were stimulated with DNP-HSA (Sigma-Aldrich) in stimulation medium (RPMI 1640 medium, 0.1% BSA, 100mM HEPES) at 37°C followed by 5 minutes incubation on ice to stop the reaction. The supernatant was collected after centrifugation and the ELISA was performed using standard procedures with the following kits and antibodies. For IL-6, the DuoSet IL-6 Kit (R&D Systems) or the coating antibody (Pharmingen #554400) and detection antibody (B&D Pharmingen #554402) were used. For TNF the coating antibody (R&D Systems #AF-410-NA) and detection antibody (R&D Systems #BAF410) were used.

Transplantation of BCR::ABL1 transduced cells

C57Bl/6 $Cpa3^{cre/+}$ or C57Bl/6 $Cpa3^{+/+}$ mice were bred in house and CD57Bl/6J recipients were obtained from Jackson Laboratory. For transplantation of BCR::ABL1 transduced cells, donors were treated using 150mg/kg 5FU i.p. 4 days prior to isolation of BM cells. Retroviral transduction was performed on 2 consecutive days, using the MSCV-IRES-GFP vector and RetroNectin reagent (Takara Bio). Recipients were irradiated (2 × 4.9 Gy) followed by transplantation of 5.5x105 cells per animal. $1x10^5$ AKC-lysed CD57Bl/6J BM cells were co-injected. Cotrimoxazol was added to the drinking water for 2 weeks after transplantation. Animal experimentation was approved by local authorities (LANUV NRW: AZ 81-02-04-2021-A016).

Mast cell staining in human bone marrow samples

Sections of formalin fixed, paraffin embedded BM samples were rehydrated followed by immunohistochemical staining in a full automated stainer (Omnis-Stainer, Agilent, DAKO, Santa Clara, CA, USA). The staining was performed using standard protocol for Flex-Staining-Kit (Agilent, DAKO) including antigen-retrieval, primary and secondary antibody, linker and DAB chromogen staining. After dehydration, sections were coverslipped and analyzed by a seasoned pathologist (T.Bra.). Used primary antibody for mast cell tryptase was clone AA1 (RTU; Agilent, DAKO).

Determination of serum tryptase in patients

Serum tryptase levels were measured in patients' sera using a fluoroimmunoenzymatic assay on a Phadia 250 analyzer (ImmunoCAP Tryptase; Thermo Fisher Scientific, Uppsala, Sweden).

Data analysis and statistics

Statistical analysis was performed using GraphPad Prism 9 and one- or two-way ANOVA were performed as described in the figure legends. Data are presented as mean ± SEM and p values < 0.05 were considered statistically significant (* $P < 0.05$; ** $P < 0.01$; *** $P < 0.001$; **** $P < 0.0001$).

Supplemental Figures and Tables

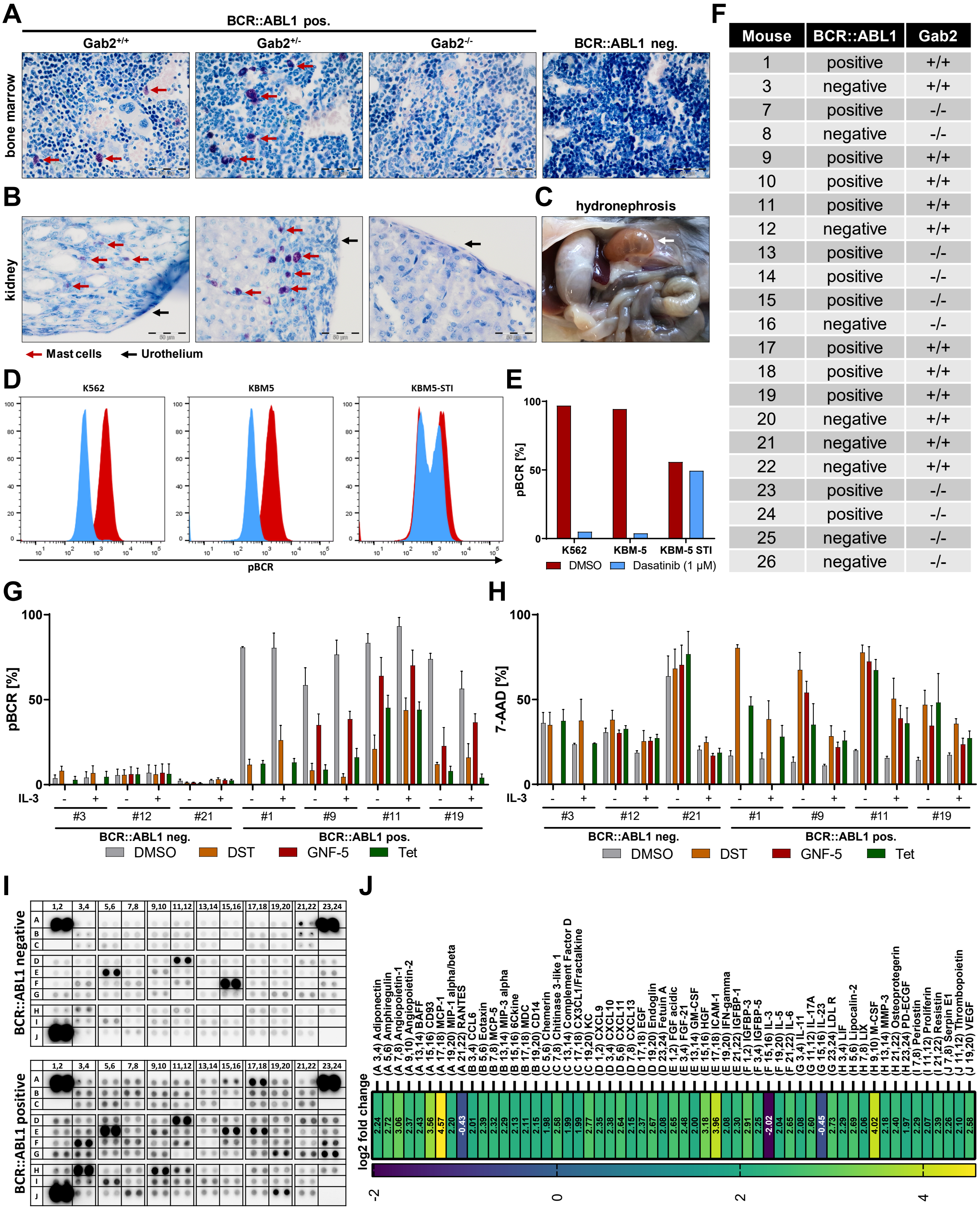

Mouse	BCR::ABL1	Gab2
1	positive	+/+
3	negative	+/+
7	positive	-/-
8	negative	-/-
9	positive	+/+
10	positive	+/+
11	positive	+/+
12	negative	+/+
13	positive	-/-
14	positive	-/-
15	positive	-/-
16	negative	-/-
17	positive	+/+
18	positive	+/+
19	positive	+/+
20	negative	+/+
21	negative	+/+
22	negative	+/+
23	positive	-/-
24	positive	-/-
25	negative	-/-
26	negative	-/-

Figure S1:
(A,B,C) BM from *ScltTA/TRE-BCR::ABL1* mice with different *Gab2* genotypes was transplanted into myeloablative irradiated C57BL/6N mice (n=3, genotype) and analyzed 200 days after transplantation. Giemsa stainings of BM (A) and kidney (B) sections. MCs (red arrows) and urothelium (black arrow) are highlighted. (C) Exemplary picture of a unilateral developed hydronephrosis (white arrow) from a mice transplanted with BM from a BCR::ABL1 expressing *Gab2*$^{+/+}$ mice. Prevalence in BCR::ABL1 positive mice: *Gab2*$^{+/+}$: 2/3 mice; *Gab2*$^{+/-}$: 1/3 mice; *Gab2*$^{-/-}$: 0/3 mice. **(D,E)** Establishment of the pBCR staining using the cell lines K562 and KBM-5, KBM5-STI (BCR::ABL1 T315I positive, TKI resistant). Cells were exposed to 1µM Dasatinib or DMSO for 1h and analyzed by flow cytometry **(F)** List of mice. **(G,H)** BCR::ABL1 positive or negative BMMCs were cultivated in standard or IL-3 containing medium and exposed to the indicated inhibitors (DST=Dasatinib, 1µM; GNF-5, 5µM) or tetracycline (Tet, 1µg/ml) for 4d and analyzed by flow cytometry. Shown is the mean of three independent performed experiments. (G) Intracellular staining of pBCR. (H). Cell viability was assessed by 7-AAD **(I,J)** (I)Total cell lysates from BCR::ABL1 positive (mouse #1) or negative (mouse #3) mice were subjected to the Proteome Profiler Mouse XL Cytokine Array (R&D Systems). (J) Log2 fold change of selected analytes after quantification All cytokines are listed in Supplementary Table S1.

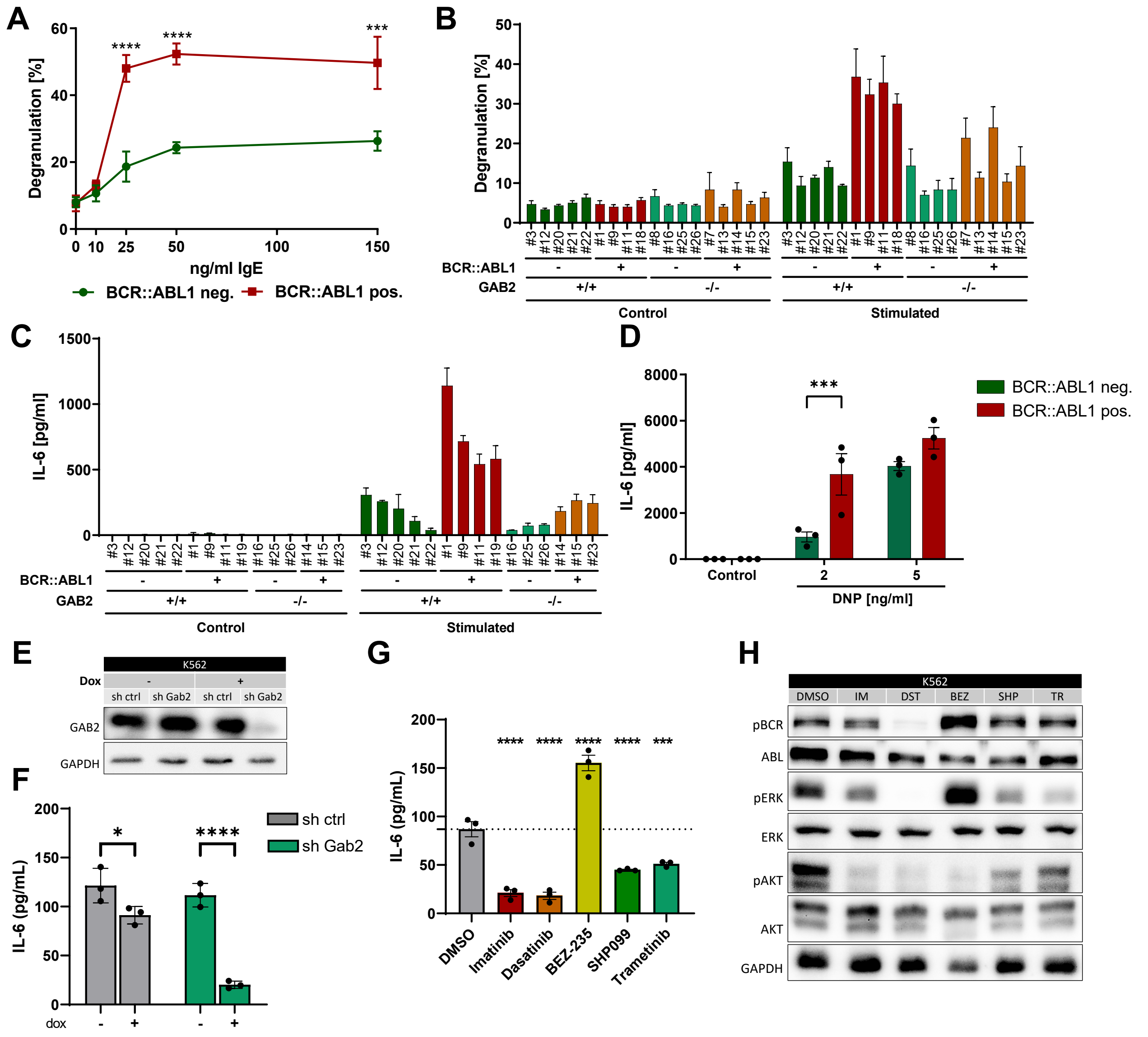

Figure S2:

(A) Degranulation of BMMCs either BCR::ABL1 positive (mouse #1) or negative (mouse #3) was assessed by β-hexosaminidase assay after loading the BMMCs with the indicated concentrations of anti-DNP-HSA overnight and stimulation with 5 ng/ml DNP-HSA **(B)** BMMCs either BCR::ABL1 positive or negative and Gab2$^{+/+}$ or Gab2$^{-/-}$ were cultivated in IL-3 containing medium. Cells were left untreated or loaded with 50 ng/ml anti-DNP-HSA IgE overnight and stimulated with 5 ng/ml DNP-HSA. The degranulation was assessed by β-hexosaminidase activity. Shown is the mean of three independent performed experiments. **(C)** BMMCs either BCR::ABL1 positive or negative and Gab2$^{+/+}$ or Gab2$^{-/-}$ were cultivated in IL-3 containing medium. Cells were loaded with 150 ng/ml IgE overnight and either left unstimulated as control or stimulated with 5 ng/ml DNP-HSA antigen. The secretion of IL-6 was assessed by ELISA. Shown is the mean of three independent performed experiments. **(D)** BCR::ABL1 negative (neg.) and positive (pos.) BMMCs were loaded with 150 ng/ml DNP-specific IgE overnight and either left unstimulated (Control) or stimulated with 2 and 5 ng/ml DNP-HSA. The amount of secreted IL-6 was measured using an ELISA. The data are presented as means ± SEM of n = 3 separate experiments conducted in triplicates. **(E-F)** K562 cells harboring dox inducible shRNA expression cassettes were treated for three days with dox, subjected to a Western blot analysis confirming successful GAB2 depletion (E) and an IL-6 ELISA (F). **(G-H)** K562 cells were treated with the indicated Inhibitors for 23h (Imatinib 1μM, Dasatinib 100nM, BEZ235 2.5μM, SHP099 5μM, Trametinib 5nM) and analyzed by Western Blot (H) and IL-6 secretion was assessed by ELISA (G) All statistics were performed using a one-way or two-way ANOVA (Fisher's LSD test) and relevant statistically significant effects are indicated by asterisks.

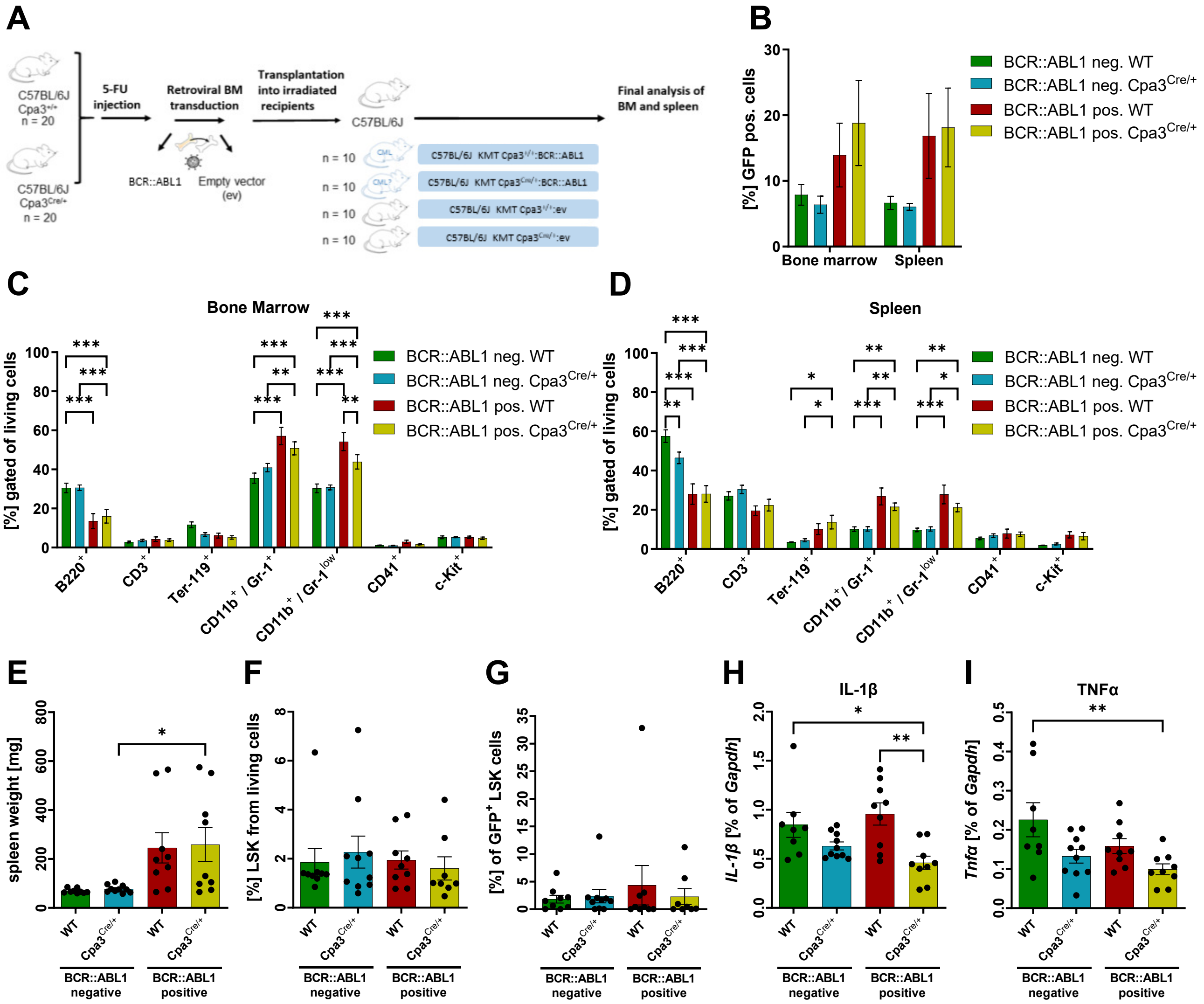

Figure S3:

(A) Schematic overview showing the design of the animal experiment **(B)** Percentage of GFP positive cells in BM and spleen determined by flow cytometry. **(C)** Composition of BM cells assessed by flow cytometry for the indicated markers **(D)** Composition of spleen cells assessed by flow cytometry for the indicated markers **(E)** Spleen weight of wild type (WT) and Cpa3Cre/+ mice transduced with empty vector (ev) control or BCR::ABL1 retrovirus **(F)** Percentage of LSK (lin-;c-kit+;Sca-1+) cells **(G)** Percentage of GFP positive cells within the LSK cell compartment **(H-I)** mRNA expression of IL-1β and Tnf was quantified by qRT-PCR of total BM cells and is shown as relative to Gapdh.

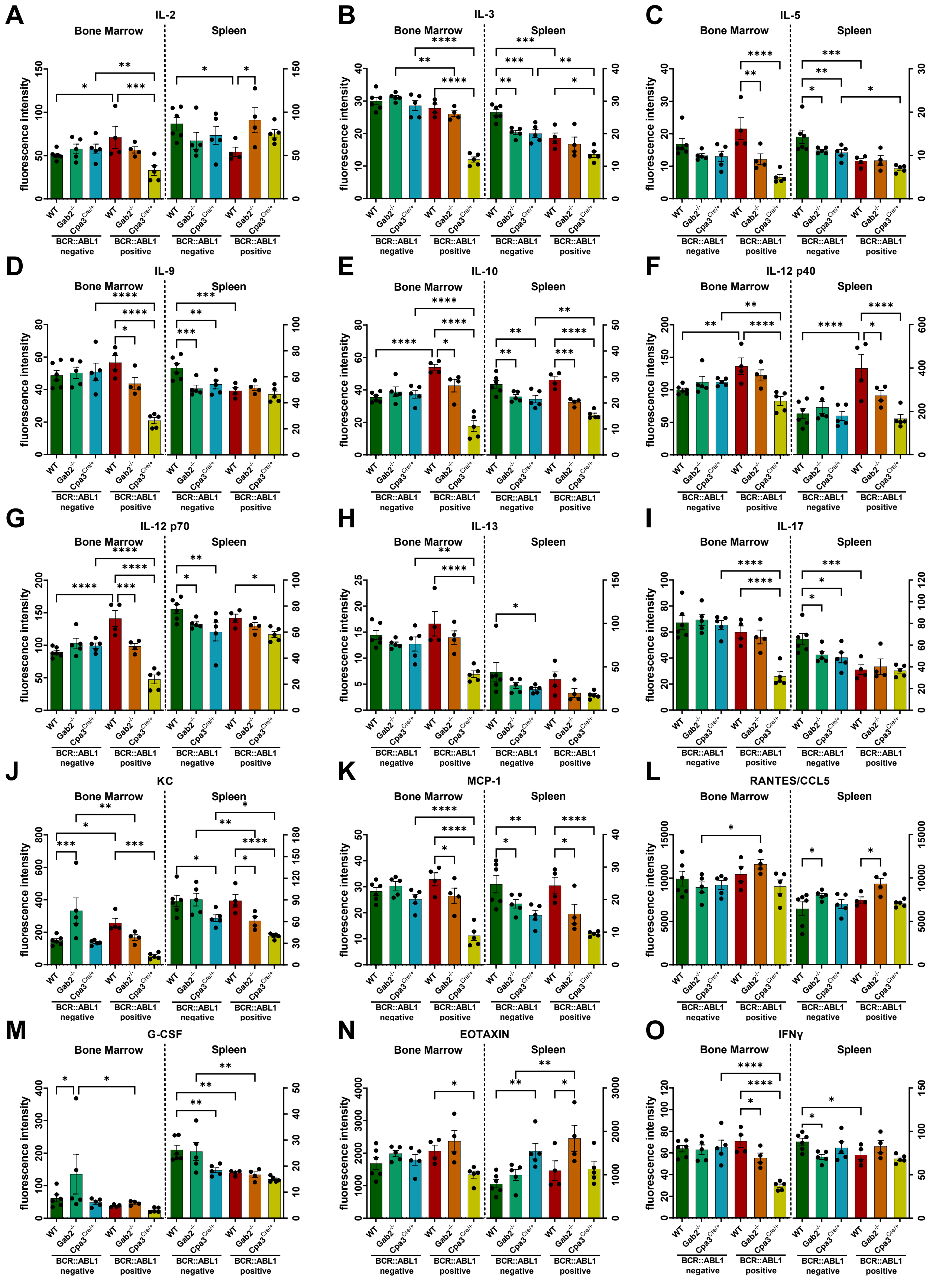

Figure S4:
(A-O) Total cell lysates from BM and spleen were subjected to a multiplex cytokine analysis. Each dot represents the biopsy of one individual mouse. All statistics were performed using a one-way ANOVA (Fisher's LSD test) and relevant statistically significant effects are indicated by asterisks.

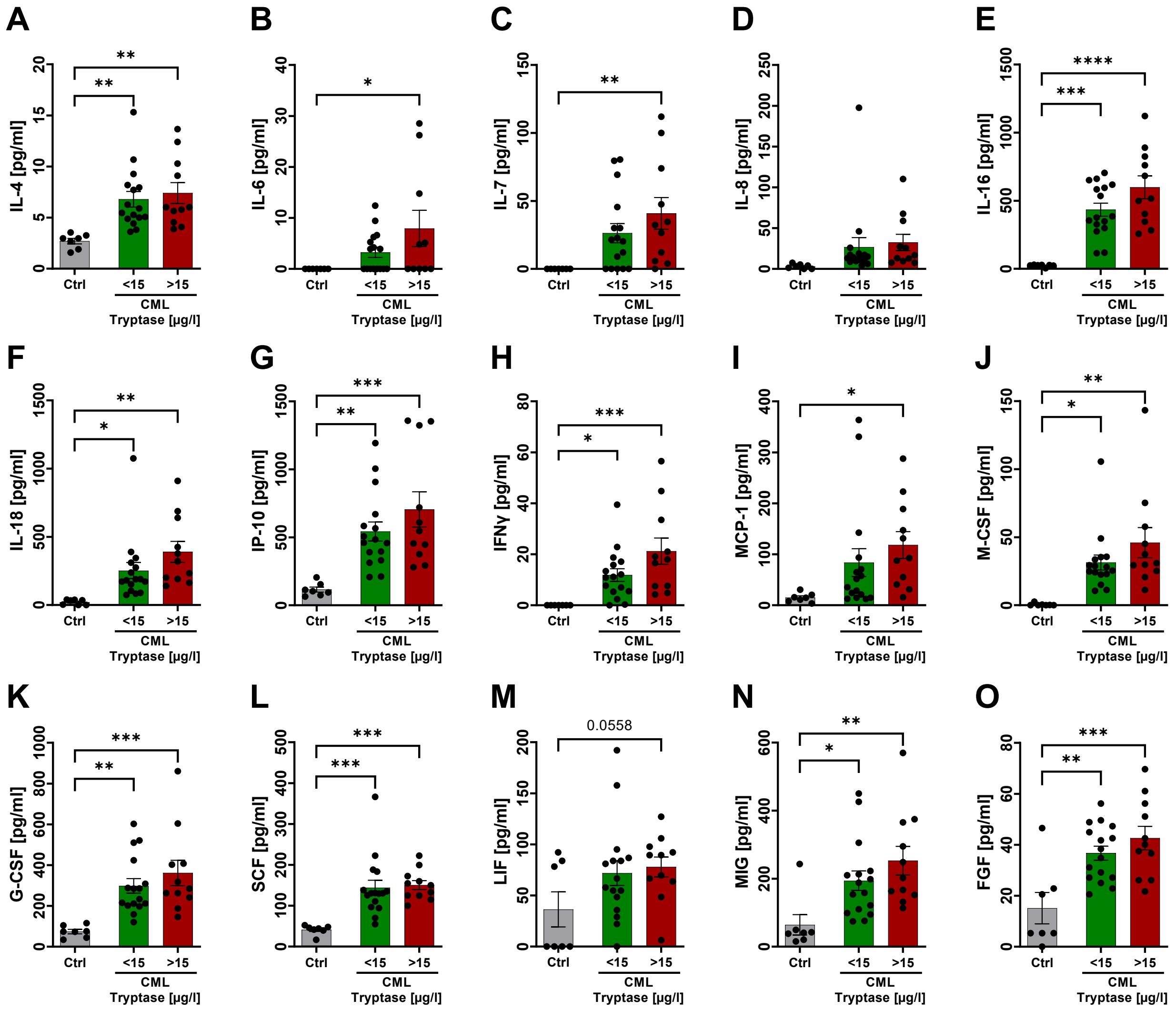

Figure S5:
(A-O) Serum samples from CML patients at diagnosis and healthy controls were subjected to a multiplex cytokine analysis. Each dot represents the sample of one individual. All statistics were performed using a one-way ANOVA (Fisher's LSD test) and relevant statistically significant effects are indicated by asterisks.

Table S1: Cytokine array

Position	Cytokine	Log2 fold change
**A1-A2**	Reference Spots	0.00
**A3-A4**	Adiponectin	2.24
**A5-A6**	Amphiregulin	2.72
**A7-A8**	Angiopoietin-1	3.06
**A9-A10**	Angiopoietin-2	2.37
**A11-A12**	Angiopoietin-like 3	1.81
**A13-A14**	BAFF	2.43
**A15-A16**	CD93	3.56
**A17-A18**	MCP-1	4.57
**A19-A20**	MIP-1 alpha/beta	2.20
**A21-A22**	RANTES/CCL5	-0.43
**A23-A24**	Reference Spots	0.03
**B3-B4**	CCL6	2.01
**B5-B6**	Eotaxin	2.39
**B7-B8**	MCP-5	2.32
**B9-B10**	TARC	1.80
**B11-B12**	MIP-3 beta	1.52
**B13-B14**	MIP-3 alpha	2.29
**B15-B16**	6Ckine	2.13
**B17-B18**	MDC	2.11
**B19-B20**	CD14	2.15
**B21-B22**	CD40	0.42
**C3-C4**	CD160	1.79
**C5-C6**	Chemerin	1.98
**C7-C8**	Chitinase 3-like 1	2.58
**C9-C10**	Coagulation Factor III	1.94
**C11-C12**	Complement Component C5/C5a	1.79
**C13-C14**	Complement Factor D	1.99
**C17-C18**	CX3CL1/Fractalkine	1.99
**C19-C20**	KC	2.77
**C21-C22**	MIP-2	1.71
**D1-D2**	CXCL9	2.35
**D3-D4**	CXCL10	2.38
**D5-D6**	CXCL11	2.64
**D7-D8**	CXCL13	2.15
**D9-D10**	CXCL16	1.73
**D11-D12**	Cystatin C	0.59
**D13-D14**	DKK-1	1.50
**D15-D16**	DPPIV	1.90
**D17-D18**	EGF	2.37
**D19-D20**	Endoglin	2.67
**D21-D22**	Endostatin	1.80
**D23-D24**	Fetuin A	2.08
**E1-E2**	FGF acidic	2.65
**E3-E4**	FGF-21	2.48
**E5-E6**	Flt-3 Ligand	0.75
**E7-E8**	Gas 6	1.81
**E9-E10**	G-CSF	1.25
**E11-E12**	GDF-15	1.83
**E13-E14**	GM-CSF	2.00
**E15-E16**	HGF	3.18
**E17-E18**	ICAM-1	3.96
**E19-E20**	IFN-gamma	2.08
**E21-E22**	IGFBP-1	2.30
**E23-E24**	IGFBP-2	0.68
**F1-F2**	IGFBP-3	2.91
**F3-F4**	IGFBP-5	2.25
**F5-F6**	IGFBP-6	1.65
**F7-F8**	IL-1 alpha	1.29
**F9-F10**	IL-1 beta	1.83

Position	Cytokine	Log2 fold change
**F11-F12**	IL-1ra/IL-1F3	1.15
**F13-F14**	IL-2	0.56
**F15-F16**	IL-3	-2.02
**F17-F18**	IL-4	1.84
**F19-F20**	IL-5	2.04
**F21-F22**	IL-6	2.65
**F23-F24**	IL-7	1.76
**G1-G2**	IL-10	1.60
**G3-G4**	IL-11	2.08
**G5-G6**	IL-12p40	1.45
**G7-G8**	IL-13	1.20
**G9-G10**	IL-15	1.25
**G11-G12**	IL-17A	2.60
**G13-G14**	IL-22	0.93
**G15-G16**	IL-23	-0.45
**G17-G18**	IL-27p28	1.54
**G19-G20**	IL-28	1.52
**G21-G22**	IL-33	1.17
**G23-G24**	LDL R	2.73
**H1-H2**	Leptin	1.67
**H3-H4**	LIF	2.29
**H5-H6**	Lipocalin-2	2.69
**H7-H8**	LIX	2.06
**H9-H10**	M-CSF	4.02
**H11-H12**	MMP-2	1.63
**H13-H14**	MMP-3	2.18
**H15-H16**	MMP-9	1.92
**H17-H18**	Myeloperoxidase	1.02
**H19-H20**	Osteopontin	0.90
**H21-H22**	Osteoprotegerin	2.40
**H23-H24**	PD-ECGF	1.97
**I1-I2**	PDGF-BB	1.07
**I3-I4**	Pentraxin 2	1.77
**I5-I6**	Pentraxin 3	1.47
**I7-I8**	Periostin	2.29
**I9-I10**	Pref-1	1.89
**I11-I12**	Proliferin	2.07
**I13-I14**	PCSK9	1.54
**I15-I16**	RAGE	1.75
**I17-I18**	RBP4	1.40
**I19-I20**	Reg3G	1.38
**I21-I22**	Resistin	2.39
**J1-J2**	Reference Spots	-0.02
**J3-J4**	E-Selectin	1.29
**J5-J6**	P-Selectin	1.91
**J7-J8**	Serpin E1	2.26
**J9-J10**	Serpin F1	1.91
**J11-J12**	Thrombopoietin	2.10
**J13-J14**	TIM-1	1.39
**J15-J16**	TNF	1.62
**J17-J18**	VCAM-1	1.35
**J19-J20**	VEGF	2.58
**J21-J22**	WISP-1	1.91

Table S2: Patient data University Hospital Aachen

Nr	Gender	Age at diagnosis [years]	Initial diagnosis	Spleen >12.5cm	Mast cells in BM [per HPF]
**2**	m	40	CML AP	yes	15
**3**	f	60	CML CP	yes	36
**4**	m	51	CML CP	yes	22
**5**	m	54	CML AP	yes	6
**15**	f	57	CML CP	no	12
**19**	m	63	CML CP	yes	25
**28**	f	45	CML CP	no	2
**46**	m	45	CML CP	yes	11
**50**	m	64	CML CP	no	1
**54**	f	58	CML CP	no	2
**58**	m	27	CML CP	yes	12
**63**	f	62	CML CP	no	20
**74**	m	59	CML CP	no	14
**76**	m	42	CML CP	no	16
**77**	f	53	CML CP	yes	3
**78**	m	43	CML CP	no	10
**83**	f	21	CML CP	yes	25
**84**	m	39	CML CP	yes	13
**89**	m	39	CML CP	no	3
**92**	m	70	CML CP	no	8

CP = Chronic Phase; AP = Accelerated Phase

Table S3: Patient data University Hospital Freiburg

Nr	Gender	Age at diagnosis [years]	Initial diagnosis	Therapy	Therapy response 3 months BCR::ABL1 [IS, %]	Spleen >12.5cm	Tryptase at diagnosis [µg/l]
**1**	f	25	CML CP	1. Imatinib<br>2. allo-HSCT	>10	yes	16.1
**2**	m	24	CML CP	1. Dasatinib	<10	yes	11.1
**3**	m	71	CML CP	1. Imatinib	>10	no	24.7
**4**	m	33	CML AP/BP	1. Dasatinib<br>2. allo-HSCT	>10	yes	44.1
**5**	f	59	CML CP	1. Imatinib	n.a.	yes	5.23
**6**	m	33	CML BP	1. Dasatinib	>10	yes	37.6
**7**	m	45	CML CP	1. Dasatinib	<10	no	10.1
**8**	m	50	CML CP	1. Dasatinib<br>2. Imatinib<br>3. allo-HSCT	>10	yes	32.8
**9**	m	74	CML CP	1. Hydroxyurea	>10<br>(no TKI → excluded from therapy analysis)	no	5.57
**10**	f	44	CML CP	1. Dasatinib<br>2. Bosutinib<br>3. Imatinib<br>4. Bosutinib	<10	yes	9.07
**11**	m	38	CML CP	1. Dasatinib	<10	yes	10.7
**12**	f	50	CML CP	1. Dasatinib<br>2. Imatinib	<10	yes	19.9
**13**	m	69	CML CP	1. Imatinib	<10	yes	11.7
**14**	f	50	CML CP	1. Dasatinib	<10	no	9.07
**15**	m	48	CML CP	1. Nilotinib + Asciminib	>10	yes	32
**16**	f	39	CML CP	1. Imatinib<br>2. Interferon<br>3. Nilotinib<br>4. Imatinib<br>5. Dasatinib<br>6. allo-HSCT	3 months: no data<br>6 months: 73%<br>→ included in analysis	yes	57.5
**17**	f	67	CML CP	1. Dasatinib	<10	no	25.7
**18**	m	29	CML CP	1. Imatinib	>10	yes	14.5
**19**	m	78	CML CP	1. Imatinib<br>2. Dasatinib<br>3. Imatinib	<10	no	10.5
**20**	m	72	CML CP	1. Dasatinib	>10	yes	13.5
**21**	f	59	CML CP	1. Dasatinib	<10	yes	2.07
**22**	m	41	CML CP	1. Dasatinib<br>2. Nilotinib<br>3. allo-HSCT	>10	yes	32.7
**23**	m	58	CML CP	1. Dasatinib	n.a.	no	6.31
**24**	m	58	CML CP	1. Nilotinib	<10	yes	7.09
**25**	m	73	CML CP	1. Imatinib	<10	yes	47.6
**26**	m	38	CML CP	1. Imatinib<br>2. Dasatinib<br>3. Nilotinib	n.a.	yes	7.94
**27**	m	71	CML CP	1. Nilotinib	<10	yes	3.33
**28**	f	40	control	-	-	-	-
**29**	f	26	control	-	-	-	-
**30**	f	24	control	-	-	-	-
**31**	f	30	control	-	-	-	-
**32**	f	23	control	-	-	-	-
**33**	m	38	control	-	-	-	-
**34**	m	27	control	-	-	-	-

CP = Chronic Phase; AP = Accelerated Phase; BP = Blast Phase

References

1. Mufti GJ, Flandrin G, Schaefer H-E, Sandberg AA, Kanfer EJ. *An Atlas of Malignant Haematology*, vol. 1 Acute Leukaemia. CRC Press, 1996.
